# Long-term outcomes in non-CAH 46,XX DSD

**DOI:** 10.3389/fendo.2024.1372887

**Published:** 2024-04-30

**Authors:** Virginie Grouthier, Anne Bachelot

**Affiliations:** ^1^ Department of Endocrinology, Diabetes and Nutrition, Centre Hospitalier Universitaire de Bordeaux, Haut Leveque Hospital, Bordeaux, France; ^2^ Univ. Bordeaux, Inserm U1034, Biology of Cardiovascular Diseases, Pessac, France; ^3^ AP-HP, Pitié-Salpêtrière Hospital, IE3M, and Centre de Référence des Maladies Endocriniennes Rares de la Croissance, and Centre de Référence des Pathologies Gynécologiques Rares, Department of Endocrinology and Reproductive Medicine, Sorbonne Université, Paris, France; ^4^ Sorbonne Université Médecine, Paris, France

**Keywords:** disorders of sex development, non-CAH 46,XX DSD, quality of life, gender identity, fertility, sexuality, follow-up

## Abstract

Differences/disorders of sex development (DSD) comprise a large group of rare congenital conditions. 46,XX DSD, excluding congenital adrenal hyperplasia (CAH), represent only a small number of these diseases. Due to the rarity of non-CAH 46,XX DSD, data on this sex chromosomal aberration were confined to case reports or case series with small numbers of patients. As the literature is still relatively sparse, medical data on the long-term effects of these pathologies remain scarce. In this review, we aim to provide an overview of current data on the long-term follow-up of patients with non-CAH 46,XX DSD, by covering the following topics: quality of life, gender identity, fertility and sexuality, global health, bone and cardiometabolic effects, cancer risk, and mortality. As non-CAH 46,XX DSD is a very rare condition, we have no accurate data on adult QoL assessment for these patients. Various factors may contribute to a legitimate questioning about their gender identity, which may differ from their sex assigned at birth. A significant proportion of gender dysphoria has been reported in various series of 46,XX DSD patients. However, it is difficult to give an accurate prevalence of gender dysphoria and gender reassignment in non-CAH 46,XX DSD because of the rarity of the data. Whatever the aetiology of non-CAH 46,XX DSD, fertility seems to be impaired. On the other hand, sexuality appears preserved in 46,XX men, whereas it is impaired in women with MRKH syndrome before treatment. Although there is still a paucity of data on general health, bone and cardiometabolic effects, and mortality, it would appear that the 46,XX DSD condition is less severely affected than other DSD conditions. Further structured and continued multi-center follow-up is needed to provide more information on the long-term outcome of this very rare non-CAH 46,XX DSD condition.

## Introduction

Differences/disorders of sex development (DSD) comprise a large group of rare congenital conditions with the incongruence of chromosomal, gonadal, and phenotypic sex (1). Therefore, DSD affects human sex determination and/or differentiation. The current medical classification is based on the genetic status of the patient. Since 2005, the new Chicago classification has subdivided DSD into three categories, as described in **Table 1** (1). Although these rare diseases are grouped under the medical umbrella term DSD, they have their own specific characteristics (incidence, context of diagnosis, phenotype, care management, short- and long-term outcomes, etc.).

**Table 1:** The current DSD classification based on the new Chicago classification.

DSD classification		
DSD with atypical sex chromosome conditions	46,XY DSD	46,XX DSD
**Turner syndrome and variants** (45,X and 45X/46,XX) **Klinefelter syndrome and variants** (47,XXY) **Other conditions with 46,XX or 46,XY karyotypes**	**46,XY karyotype with gonadal development disorders** (pure or partial gonadal dysgenesis, ovotestiscular DSD, ovarian DSD, vanishing testis) **46,XY karyotype and disturbances of testosterone synthesis** (StAR deficiency, 3β-hydroxysteroid dehydrogenase deficiency, 17α-hydroxylase/17,20-lyase deficiency, 17β-hydroxysteroid dehydrogenase deficiency, 5α-reductase deficiency, POR deficiency, AMH mutation, LHCGR mutation and insensitivity to LH) **46,XY karyotype and disturbances of testosterone action** (complete androgen insensitivity syndrome) **46,XY karyotype and complex malformation syndromes**	**46,XX karyotype with androgen excess** (congenital adrenal hyperplasia with 21 hydroxylase deficiency, 3 β-hydroxysteroid dehydrogenase deficiency, 11 β-hydroxylase deficiency, POR deficiency, aromatase deficiency, exogenous causes) **46,XX karyotype with gonadal development disorders** (complete or partial gonadal dysgenesis, ovotesticular DSD, 46XX males, monogenic forms of premature ovarian insufficiency) **46,XX karyotype and non-development of uterus or vagina** (Mayer–Rokitansky–Küster–Hauser (MRKH) syndrome and other complex malformation syndromes)

The rarity and complexity of these various conditions, combined with the lost follow-up of patients, make studies including meaningful numbers of adults living with a DSD a real challenge. Currently, too little is known about these rare diseases, especially about their long-term outcomes in adults. In recent years, international registries have been established to provide structured and continuous monitoring. For instance, a European multidisciplinary consortium called dsd-LIFE started in 2012 with the aim of evaluating and improving the treatment and care of patients with DSD (2). To date, it collected information from 1,040 persons with DSD conditions in six countries (Germany, France, the Netherlands, Poland, Sweden, and the UK). Another example is the Empower-DSD project, which is a prospective, longitudinal, and noncontrolled multicenter study led in Germany with the main aim of developing, implementing, and evaluating an age-appropriate educational program for 700 children and young people with DSD (3).

Among the 46,XX DSD patients, the large majority have DSD with androgen excess and, more specifically, congenital adrenal hyperplasia (CAH) (4–6). In the dsd-LIFE cohort of 1,040 individuals with DSD, 253 have 46,XX DSD, and of these, 226 (89.3%) have CAH, only 21 (8.3%) have 46,XX gonadal dysgenesis, and six (2.4%) are 46,XX men (2). It is important to specify that patients with Mayer–Rokitansky–Küster–Hauser (MRKH) syndrome were not included in this cohort. From a nationwide registry study including 41 men with a verified diagnosis of DSD 46,XX, the prevalence of non-CAH 46,XX DSD men in Denmark was estimated at 3.5–4.7 per 100,000 (7). Due to the rarity of non-CAH 46,XX DSD, the literature on this sex chromosomal aberration was limited to case reports or case series with small numbers of patients. This review is specifically concerned with non-CAH 46,XX DSD, including other disorders of androgen excess such as aromatase deficiency with CYP19A1 mutation, disorders of gonadal development (ovotesticular DSD, 46XX men or 46,XX testicular DSD, and monogenic forms of primary ovarian insufficiency with mutations in genes involved in ovarian development), and unclassified disorders with MRKH syndrome (**Table 2**). We will not discuss here the iatrogenic causes of androgen excess or luteoma.

**Table 2:** Description of the main aetiologies of non-CAH 46,XX DSD.

	Sex assignment at birth	Prevalence	Clinical description/phenotype	Hormonal patterns	Main candidate genes	Impact on fertility	Prognosis
***46,XX male or 46,XX testicular DSD***	Men	1:20,000 newborn boys	Phenotypically male characteristics, small testis at puberty Hypospadias, cryptorchidy (SRY−)	Testosterone ↔ FSH ↗ LH ↗ Inhibin B ↘	SRY SOX9 DAX1 NR5A1 WNT4	↘↘ with azoospermia	Normal life expectancy ± hormone replacement therapy
***46,XX ovotestis***	Men or women depending on gonad histology	1:20,000 births	Atypical genitalia is often diagnosed at the neonatal period (external genitalia ranges from apparent woman to man with hypospadias or isolated bilaterally undescended ovotestes) Most affected individuals have female internal genitalia (uterus, hemi-uterus, or rudimentary uterus)	Depending on gonad histology	SRY SOX9 SOX3 NROB1 NR5A1	↘ in men ↘ women but someone have some potential for fertility	Normal life expectancy ± hormone replacement therapy
***46,XX DSD due to aromatase deficiency***	Women	?	Atypical genitalia and virilization in women, then primary amenorrhea and no pubertal growth spurt and breast development. Tall stature and osteoporosis	Estradiol ↘ Testosterone↗	CYP19A1	Fertility potential still unknown	Hormone replacement therapy
***Monogenic forms of POI***	Women	2-4:100 before age 40 1:1,000 before age 30 1:10,000 before age 20	Frequently amenorrhea and impuberism	Estradiol ↘ FSH ↗ LH ↗ AMH ↘	FSH-R FOXL2 NR5A1 GDF9 BMP15	↘	Normal life expectancy + hormone replacement therapy
***MRHK syndrome***	Women	1:4,500	Phenotypically female characteristics in type I (isolated uterovaginal aplasia) Renal, skeletal, hearing, and cardiac abnormalities in type II (MURC Syndrome = Mullerian, renal, cervicothoracic somite syndrome)	Generally normal gonadal axis	Mostly unknown Some cases WNT4?	↘	Normal life expectancy No hormone replacement therapy

↔ means stability ; ↑ menas increase, ↓ means decrease, ? means no data to conclude.

The management of DSD is complex, and so are the long-term consequences, particularly in terms of treatment options, fertility, or even quality of life. As the literature is still relatively sparse, medical data on the long-term effects of these pathologies remain scarce. In addition, the cohorts are generally limited to a small number of pediatric patients, with short-term follow-up, which explains why data on the aging of these populations are still limited. To our knowledge, there is no specific data on the transition of pediatric to adult care for non-CAH 46,XX DSD patients. Many of them are lost from sight in adulthood. The first lesson to be learned from this review will therefore be the importance of continuing to follow up this population over the longer term, especially to study the occurrence of rare medical events associated with aging, such as the risk of psychological impact, cardiometabolic disease, or cancer. Nevertheless, in this review, we provide an overview of current data on the long-term follow-up of patients with non-CAH 46,XX DSD. We will review the following topics: quality of life, gender identity, fertility and sexuality, global health, bone and cardiometabolic impacts, cancer risk, and mortality of non-CAH 46,XX DSD.

## Quality of life

The World Health Organization defined quality of life (QoL) as “an individual’s perception of their position in life in the context of the culture and value systems in which they live, and in relation to their goals, expectations, standards, and concerns”. The measurement represents people’s individual perceptions about their position relative to other people and relative to their own expectations (8).

Because non-CAH 46,XX DSD is a very rare condition, we are not aware of any precise data on the assessment of QoL in adulthood in these patients.

In 1999, a pilot survey looked at the QoL of 10 DSD adults with a standardized quality-of-life survey specific to this population (9). All considered themselves healthy and physically active, and nine were satisfied with their social life, whereas one was dissatisfied. This paper is one of the first to focus on the QoL of DSD patients, although the lack of karyotype precision is a major limitation. In 2017, Bennecke et al. assessed health-related QoL using a validated survey (the Short Form (SF-36) Health Survey and the Brief Symptom Inventory) from 110 adults with DSD, aged between 17 and 62 years (10). Only three persons were non-CAH 46,XX DSD patients (one with aromatase deficiency, one with ovarian insufficiency, and one with complete gonadal dysgenesis) included in a group of eight people defined as “individuals with DSD conditions and other gender groups”. The mean age of this group was 33.18 years (range 22–49). There was no significant difference in physical health-related QoL between them and controls. Even if all participants in the cohort reported an alteration in psychological health-related QoL, the difference failed to reach significance within the subgroup analyses. Recently, the dsd-LIFE cohort, which includes a group of 27 patients with non-CAH 46,XX DSD, has analyzed the QoL of DSD patients (11). Poor or fair health was reported by 367 participants (35.3%), and 479 participants (46.1%) had another additional longstanding illness in addition to their DSD condition. The physical and also psychological health-related QoL of DSD patients was lower than that of the healthy reference population. The dsd-LIFE cohort participants in each country scored much lower in their social relationships than the healthy reference population. Unfortunately, there was no data specific to non-CAH 46,XX DSD patients, which represent only 2.6% (27/1,040) of this European cohort.

It is therefore difficult to draw any conclusions about the QoL of people with non-CAH 46,XX DSD, given the paucity of the literature and the rarity of this karyotypic condition.

On the other hand, the literature seems to be a little more extensive and precise on the QoL of women with MRKH syndrome. A recent systematic review aimed to evaluate the long-lasting impact of MRKH diagnosis among adolescent girls before and after any therapeutic intervention in comparison with healthy women (12). Ten studies including 518 patients examined psychological and psychosocial outcomes using different validated questionnaires. Various studies have shown a higher prevalence of anxiety and depression symptomatology, eating disorders, and lower self-esteem in patients with MRKH. However, similar results were not found in all studies, highlighting the great heterogeneity of the studies, which may be due to differences between participants (age, surgical or medical treatment, etc.). Six studies, including 312 patients, had examined QoL outcomes using principally WHOQOL-Bref and SF-36 Health Survey and the Mental Health Component Summary Score (MCS). Despite a clear heterogeneity in these studies, the majority reported a significant impairment of mental-health-related QoL in MRKH patients compared with the control groups. On the other hand, no significant difference was found between MRKH syndrome and poorer physical health. A French study had recently shown no difference in QoL after the creation of neovagina among patients with MRKH who could have surgical procedures or vaginal dilatation (13). Indeed, a total of 131 patients (84 with surgical procedures, 26 with dilation therapy, and 20 with intercourse) aged 26.5 years ± 5.5 years had participated in the survey. There were no significant differences in the general QoL depending on the type of management (physical health: *p* = 0.63; psychological health: *p* = 0.56; social relationship: *p* = 0.52; environment: *p* = 0.054). The vaginal length was significantly shorter after dilation therapy (9.3 cm [5.5–12]) versus after surgery (11 cm [6–15]) (*p* = 0.039), with no difference between the surgery and intercourse groups. Similar results were published very recently by Kang et al. among 88 women with MRKH treated by vaginal dilatation and 45 by surgical procedures (14). No significant differences were found in the 12-item World Health Organization Disability Assessment Schedule 2 scores between the dilation group and the surgery group (respectively 8.33 [interquartile range, 4.17–15.62] and 6.25 [2.08–14.58], *p* = 0.306). In conclusion, women with MRKH syndrome seem to have a reduced psychological QoL, regardless of management care, but their physical health does not appear to be impaired.

## Gender identity

Gender identity refers to one’s internal, deeply held sense of gender (15). Gender incongruence is an umbrella term used when the gender identity and/or gender expression differ from what is typically associated with the designated gender. Gender dysphoria is the distress and unease experienced if gender identity and designated gender are not completely congruent. Therefore, gender incongruence may lead to gender dysphoria and a desire to change gender or gender transition.

For people with DSD, their hormonal history, their karyotype, the presence of genital development variation, or even psychosocial factors may contribute to a legitimate questioning about their gender identity, which may differ from their sex assigned at birth. Indeed, DSD conditions seem to be important sources of gender changes (16–18) except Klinefelter and Turner syndromes, which seem to be less affected by gender incongruence (16). Here again, even if there is relatively abundant literature on the subject of gender identity in people with DSD, especially among CAH patients, too little data are available on the specific cohort of non-CAH 46,XX DSD.

From the European dsd-LIFE cohort, Kreukels et al. tried to assess gender change and gender dysphoria in a large sample of individuals with different DSD (16). In this cohort, 12 participants among 1,040 participants (1.15%) reported not feeling man or woman or did not experience themselves in the male–female spectrum at all (none among non-CAH 46,XX DSD). Five percent of patients (*n* = 47) had experienced a change of gender in their lives with 36 (80%) before puberty, most likely imposed by a physician, whereas nine patient-initiated gender changes occurred after puberty (five patients in the group “men with XY DSD”, two patients in the group “women with XY karyotype with androgen effects”, one patient in the group “XY with CAIS or complete gonadal dysgenesis and XX gonadal dysgenesis without androgen effects”, and one woman CAH). In a recent US study comparing young people with a diagnosis of DSD (*n* = 1,216) with matched controls (1:4), 1.1% of individuals with a DSD had a diagnosis of gender dysphoria (19), and, in comparison, data in the USA suggest that 0.6% of adults and up to 1.8% of youth identify as transgender (without necessary gender dysphoria) in the general population (19, 20). In another recent study from South Africa, 64 people with ovotesticular DSD, histologically confirmed, were assessed, treated, and followed up at the Endocrine Clinic over a period of 20 years (21). The most common karyotype was 46,XX (88%; *n* = 56), followed by 46,XY (8%), 46,XY/45,X (3%), and 46,XX/46,XY (1%). The authors highlighted a significant prevalence of gender dysphoria (*n* = 8, 11%), and five patients had benefited from a reassigned gender (four children and one adult at 20 years old). A couple of years ago, Palma Sircili et al. found the same proportion (15%) of gender dysphoria among 20 patients with ovotesticular DSD (22). A 46,XX karyotype was observed in 18 patients (90%), and two (10%) had a 46,XX/46,XY karyotype. Gender assignment at birth was boy in 13 patients and girl in seven. Three girls were later reassigned to the male sex (at ages 5, 10, and 20 years, respectively).

Because of the rarity of the non-CAH 46.XX DSD situation, it is difficult to give an accurate prevalence of gender dysphoria and gender reassignment in this population. Specialized management care with informed clinicians seems necessary to help and guide adolescents and young adults with their choices.

## Fertility and sexuality

DSD often affect the ability to have biological children. Fertility, and even more so, sexuality, becomes a priority issue in adolescence and adulthood. Non-CAH 46,XX DSD cover a range of conditions with their own impact on issues of fertility and sexuality. Indeed, although fertility is significantly reduced in DSD (except CAH), it differs between types of pathology. The presence of functional gonads and uterus should be assessed, as should patients’ knowledge of the impact of their disease on their reproductive function.

Slowikowska-Hilczer et al. studied fertility in the dsd-LIFE cohort, in which the mean age of participants was 32.4 years ± 13.6 years. When CAH were excluded, only 0.7% of the cohort were able to have children without assisted reproductive technology (ART). More precisely, of the patients with complete or partial gonadal dysgenesis, 48% to 80% had received information on fertility from doctors, and 9% to 15% were not satisfied with the information. However, 5% to 8% of them were able to have their own biological children. Between 50% and 70% of them wanted to have fertility treatment and try new fertility techniques. Finally, seven patients became parents (three thanks to egg donation, two to sperm donation, and two by adoption) (23). With regard to the men with 46,XX (six patients in the dsd-LIFe cohort, aged 41.2 years ± 11.2 years), four patients (66.7%) recalled fertility discussion with their health professional, and three were satisfied with this discussion. All of them knew they were not able to have their own biological children. Two men had children (one thanks to sperm donation and the other by adoption) (23). A number of cases of 46,XX men with infertility (azoospermia) have been regularly reported in the literature for several decades (24–29). 46,XX men generally discover their pathology because of infertility. They usually have normal male phenotypes, small testes, azoospermia, and hypergonadotropic hypogonadism (30). In a more important cohort, 144 adult men with 46,XX DSD (82.6% SRY positive and 17.4% SRY negative) were analyzed (31). All had azoospermia. In 2007, a Danish study compared the programs of 46,XX men (*n* = 7) and 47,XXY Klinefelter men (*n* = 40) with those of healthy (*n* = 2,136) and fertile men (*n* = 349) (32). All participants were between 17.9 and 43.8 years old. Median semen volume was significantly smaller in the patients when compared with healthy men and fertile men (respectively 2.0 mL [0.2–5.7], 3.1 mL [0.3–12.5], and 3.6 mL [0.6–12.5], *p* < 0.0001). However, there was no difference between semen volume in 47,XXY and 46,XX men. Azoospermia was present in all 46,XX men. Recently, the testicular architecture of men with 46,XX DSD SRY positive (*n* = 4) was compared to Klinefelter (*n* = 4) and to healthy men (*n* = 4) (33). A smaller number of tubules and more SOX9-negative but similar proportions of DMRT1-negative Sertoli cells were found in 46,XX DSD compared to healthy men. The lower number of tubules and severe Leydig cell hyperplasia observed in 46,XX DSD was similar to Klinefelter. All gonadal biopsies performed in 20 46,XX DSD men in Boucekkine et al. revealed dysgenetic and immature testes with germinal aplasia. Neither spermatogonia nor spermatozoa were found in adult testes, but Sertoli cells were clearly present (normal or hyperplasic cells) (34). Thus, azoospermia is reported to be universal in 46,XX men (35), and fertility requires ART with sperm donation.

On the other hand, regarding sexuality, 46,XX DSD men seem to have no symptoms or dysfunctions. In Yiğman’s study, 10 men with 46,XX DSD SRY positive and azoospermia were retrospectively compared to healthy men (36). The average age was 27.9 years ± 3.5 years in cases and 27.2 years ± 3.6 years in controls, and all 46,XX men had a normal masculinized phenotype. There was no statistically significant difference between the two groups in terms of erectile dysfunction evaluated by the International Index of Erectile Function questionnaire (*p* > 0.05). This result was confirmed by a recent systemic review (37 publications and 64 patients with 46,XX male syndrome aged 33.14 years ± 11.4 years): normal erectile function was present in 27/30 cases (90%) and libido was preserved in 20/20 patients (100%).

Another condition of 46,XX DSD is an ovotesticular disorder defined by the coexistence of testes and ovaries within the same individual. Their prognosis for fertility seems to depend on the histology of the gonad, although it is compromised in the majority of cases. In a recent French study, 16 patients (10 reared as women and six as men) with SRY-negative 46,XX testicular/ovotesticular DSD were followed up between 1994 and 2018. The most common gonadal histology was ovotestis (68%) followed by ovary (23%), and the less common was testis (9%) (37). Three men and two women reached adulthood. Puberty started spontaneously for the two girls, who were then menstruating cyclically and regularly. One of them became pregnant thereafter, without medically assisted reproduction. For men with gonads in the scrotal position, testicular parenchyma was found to be dysgenetic without spermatogonia in seminiferous tubules. A retrospective cohort of 20 patients with ovotesticular DSD included 18 patients with 46,XX karyotype followed up during 25 years (range 4 to 46). Puberty started spontaneously in 14 patients between the ages of 11 and 14. Ten adult patients (eight men and two women) reported sexual activity. All male patients reported orgasm, and two reported ejaculations. None had succeeded in conceiving (22). In terms of fertility, the ovarian component of the gonad tends to have a relatively normal histology, whereas the testicular component is more often dysgenetic, with limited germ cells and rarely sperm (35).

Regarding 46,XX DSD due to aromatase deficiency, another very rare condition of DSD, a recent systematic review of 43 cases highlighted a degree of heterogeneity in the phenotype associated with the mutation (truncating or nontruncating variant) (38). Although not significant, spontaneous thelarche occurred in nine of 14 (64.2%) women with nontruncating variants compared with two of eight (25%) with truncating variants (*p* = 0.1), whereas spontaneous menarche was exclusively associated with nontruncating variants (three of 14 (21.4%) vs. 0 of eight; *p* = 0.2). However, their future fertility potential is still unknown.

With regard to another DSD condition, the MRKH syndrome with absence or hypotrophy of the vagina has a direct and obvious effect on the sexuality of the patient. Treatment of vaginal aplasia, which is necessary to allow intercourse with penetration, is a real issue that needs to be addressed in these MRKH women. The management of vaginal hypoplasia is complex and multifaceted. In addition to the central role of the team psychologist, treating vaginal hypoplasia requires real expertise. Indeed, the vagina can be lengthened using non-surgical (dilation therapy) or sometimes surgical methods. Studies have compared surgical techniques with the dilation method, using validated sexual satisfaction surveys, and several reviews of the literature have already been published. The conclusion that can be drawn from all these studies is that anatomical and functional outcomes are comparable regardless of the technique chosen, but that surgery is associated with more serious morbidity (39). Actually, in the Chinese cross-sectional study, which included 88 women with MRKH syndrome treated with vaginal dilation and 45 treated by surgical procedures, no significant differences were observed in global sexual function (14). The only different dimension of the Female Sexual Function Index (FSFI) was a higher orgasm score in the dilation group. The same results were found in the French multicenter study, which included 131 adult women with MRKH (84 by surgery, 26 by dilation therapy, and 20 by intercourse) at least 1 year after completing vaginal agenesis management (13). The FSFI was not different between groups except for the satisfaction dimension, with a higher score in the intercourse group (30.2 [7.8–34.8], *p* = 0.044) compared to the surgery and dilation therapy groups (median 26 [2.8–34.8] and 24.7 [2.6–34.4], respectively, *p* = 0.044). The Female Sexual Distress Scale-Revised median scores were, respectively, 17 [0–52], 20 [0–47], and 10 [10–40] in the surgery, dilation therapy, and intercourse groups (*p* = 0.38), with sexual distress in 71% of patients. In the surgery group, 45% of women had complications, 23.8% required a second operation, and 42% needed postoperative dilation. Conversely, 50% of the dilation group needed maintenance dilation. As expected, the posttreatment sexual quality-of-life score appeared to be lower than that of the general population (39). Sexual arousal, lubrication, orgasm, and dyspareunia scores were the most affected (*p* = 0.01). Despite the scarcity of literature, all these results have led to the indication of vaginal dilation as the first-line therapy for MRKH patients, but sexuality still appears to be affected in these patients.

Moreover, women with MRKH syndrome generally have normal ovarian function. The absence of a uterus quickly brings to light the absolute infertility. Careful and sensitive counseling about fertility as well as discussing valuable alternatives, such as adoption, are paramount. However, in 2015, the first live birth after uterus transplantation in women with MRKH syndrome was reported (40). Uterine transplantation is a recent and booming area of research. It can only be considered in validated and supervised clinical research programs. The first registry of the International Society of Uterus Transplantation, published in January 2023, reported 45 transplantation procedures from 2012 to 2020 (41). The majority (78%) of those procedures were live donor transplants that had a median age of 50 years (32–62). Postoperative complications were registered in 20% of live donors and 24% of recipients. Rejection episodes were more frequent early after transplantation (months 1–5; 33%) compared with later time points (months 6–10; 21%). A total of 19 live births from 16 recipients were reported. All live births occurred after *in vitro* fertilization. Since 2020, new outcomes have been published, and to date, more than 80 uterine transplantations have been actually performed, resulting in more than 40 healthy live births. This surgical procedure, although exceptional at the moment and despite persistent ethical considerations, represents a potential therapeutic hope for patients without a uterus, such as those with MRKH syndrome.

## Global health, bone and cardiometabolic effects

Knowledge about health status in adults with DSD is scarce, especially in non-CAH 46,XX DSD because of the rarity of this condition. In the dsd-LIFE cohort, people with DSD generally reported fair to very good general health (91.4%) (42). Only 8.6% of the whole cohort reported bad or very bad general health compared with 6% of the general population. Looking specifically at the 46.XX DSD population, the figures were slightly worse. Indeed, fair to very good general health was reported by 83.3% of 46,XX DSD patients, and 16.7% reported very bad general health. However, long-standing health problems other than DSD and feeling limited in daily life were more frequently reported by DSD patients (51.0% and 38.6%, respectively) than by controls (24.5% and 13.8%, *p* < 0.0001 both), except for XX-DSD patients (43.8%, *p* = 0.15 and 21.4%, *p* = 0.45).

Regarding cardiovascular and metabolic disorders, around 15% of DSD in the dsd-LIFE cohort, had at least one cardiovascular disease (compared with 5% of controls), 3.1% had two, and 0.8% had three or more diagnoses. There were no differences between women and men. Increased cardiovascular risk factors (diabetes, hypertension, dyslipidemia) were more common in all DSD groups compared with controls, but with the exception of the XX-DSD population (42). However, it should be kept in mind that the non-CAH 46.XX DSD group was small (*n* = 21) and relatively young (32.4 years old ± 13.6 years old). In Berglund’s study, which included 44 men with 46,XX DSD, no increased risk of ischemic vascular diseases was detected, albeit half of the 46,XX DSD men within the cardiovascular diagnosis group had a diagnosis of ischemic heart disease (7). However, this may be related to a lack of statistical power.

Focusing on osteoporosis and bone fracture, no difference was found between XX-DSD and controls in the dsd-LIFE cohort (42). A Chinese team has reported retrospective data about 33 patients diagnosed with 46,XX pure gonadal dysgenesis followed up at Peking Union Medical College Hospital from January 2011 to March 2016 (43). At the time of diagnosis, the patients were 19.53 years old ± 3.60 years old (range 14–28) and had already presented osteoporosis. Individualized hormone placement therapy was initiated after diagnosis, and 18 patients (54.55%) were followed up for more than 2 years. With the treatment, all patients had obvious bone density improvement. Indeed, the incidence of osteopenia changed from 69.7% to 22.2%, and osteoporosis changed from 18.2% to 0%. Take another example of DSD, such as aromatase deficiency, a very rare DSD condition with an incidence lower than 1/100,000, which can also be responsible for osteoporosis. Bone metabolism is affected by mutations responsible for the loss of function of the aromatase protein. Indeed, estrogen deficiency due to aromatase deficiency results in altered bone homeostasis. In 2020, a review of the literature included 30 patients with 46,XX DSD due to aromatase deficiency and low bone mass found in five of eight patients (44). Most of these symptoms appear to be reversible with estrogen replacement therapy, without knowing the appropriate timing and dosage.

In the dsd-LIFE cohort, Falhammar et al. looked at other comorbidities that could appear in adulthood over the long term. Autoimmune disorders were reported in 30% of XX-DSD patients (42). There were no differences between XX-DSD and controls concerning renal and neurological disorders or visual, hearing, and urinary issues. Voice dissatisfaction was reported in most (82.4%) non-CAH 46,XX DSD patients (45).

Finally, there is still a paucity of data on long-term outcomes. However, once diagnosed and managed, the non-CAH 46,XX DSD group does not appear to be at greater risk of developing associated comorbidities.

## Cancer and mortality

Dysgenic gonads are known to be predisposed to degenerate, but the non-CAH 46,XX DSD subgroup seems to be relatively protected. A recent Danish study has analyzed morbidity and mortality in men with 46,XX DSD (7). It was a nationwide registry study including 44 men with a verified diagnosis of 46,XX DSD, born between 1908 and 2012, and followed up until 2014. Each patient was paired with a randomly selected, age-matched control group of 100 men and 100 women. Mortality was not increased (HR = 0.6, 95% CI: 0.2–2.5) compared to male controls. Moreover, overall morbidity was not increased compared to male controls when excluding endocrine and urogenital diseases as well as congenital malformations (HR = 1.2, 95% CI: 0.8–1.6). No records of breast cancer were observed, although breast cancer has been reported in men with 46,XX DSD (46, 47). In the dsd-LIFE cohort, malignancy occurred in 4.1% and was not increased in the 46,XX DSD group compared to the control group (42). A French retrospective study reviewed 28 patients with DSD between 1965 and 2005 (48). The most common karyotype was 46,XX (82% of the cohort), with SRY positive in 35% of cases. Patients were followed up for a median of 11 years (range 3 months to 35 years), with 18 (55%) being 13 years or older at the last consultation. No gonadal tumors were detected, but follow-up after puberty lasted only 2 years on average (range 1 to 14). The absence of the Y chromosome appears to be an effective protective factor. More recently, Huang et al. found dysgerminoma in one of their 33 patients with 46,XX DSD (43). Therefore, although these data are reassuring, young age of the population studied is an obvious limitation that needs to be taken into account. For non-CAH 46,XX DSD patients, accurate diagnosis and close follow-up are also strongly recommended. Finally, the risk of cancer in non-CAH 46,XXD DSD appears to be low, so prophylactic gonadectomy is not justified in these patients.

## Conclusion

Non-CAH 46,XX DSD is a very rare condition within the large spectrum of rare diseases that are DSD. To date, there is a very limited amount of data available on the long-term follow-up of this condition. Many patients are likely to be lost to follow-up in adulthood. Although psychological but also physical health-related quality of life appears to be altered in DSD patients, no specific data are available for non-CAH 46,XX DSD to date. Similarly, various factors (psychosocial, hormonal, medical history, etc.) may contribute to a legitimate questioning of gender identity, which may differ from the sex assigned at birth in people with DSD. However, there are no precise data on the accurate prevalence of gender dysphoria and gender reassignment in non-CAH 46,XX DSD people. Regarding global health, a higher proportion of patients with 46.XX DSD seems to report impaired general health. However, there does not appear to be more comorbidity in this study population, although data is still very scarce. Finally, data on the risk of malignancy in the 46,XX DSD population seems reassuring, but longer-term follow-up of this population seems necessary to confirm this.

It is therefore essential to put in place an organized transition to better understand the outcomes of these patients and improve their care. Therefore, it is important to set up multicenter and international cohorts combining different series of small numbers of patients to gain a better understanding of the natural history and aging of these patients, with a particular interest in investigating their quality of life, sexuality, and fertility. In addition, information on future cardiovascular and bone health remains limited, making the risks from these potential long-term effects difficult to assess.

## Author contributions

VG: Writing – original draft, Writing – review & editing. AB: Writing – original draft, Writing – review & editing.
